# Sub-inhibitory concentrations of fosfomycin enhance *Staphylococcus aureus* biofilm formation by a *sarA*-dependent mechanism

**DOI:** 10.1128/spectrum.01521-25

**Published:** 2025-07-21

**Authors:** Tingting Zeng, Ying Wang, Qing Zhu, Huimin Xi, Mei-fang Liu, Peng Liu, YunXue Bai, Lei Yuan, Rui Zhao, Yi-yun Sheng, Qianbin Dai

**Affiliations:** 1 Department of Clinical Laboratory, Medical Center of Burn Plastic and Wound Repair, The First Affiliated Hospital, Jiangxi Medical College, Nanchang University 47861, Nanchang, China; 2 School of Public Health, Jiangxi Medical College, Nanchang University 47861, Nanchang, China; 3 Department of Pathology, The First Affiliated Hospital, Jiangxi Medical College, Nanchang University 47861, Nanchang, China; Universidad Maimonides, Buenos Aires, Argentina

**Keywords:** *Staphylococcus aureus*, fosfomycin, biofilm, polysaccharide intercellular adhesin, *sarA*

## Abstract

*Staphylococcus aureus* readily forms biofilms, which contribute to antimicrobial resistance and the persistence of chronic infections. This study investigates the effects of sub-inhibitory concentrations of fosfomycin on *S. aureus* biofilm formation and elucidates the underlying molecular mechanisms. Using crystal violet staining and confocal laser scanning microscopy, we demonstrated that fosfomycin at 1 µg/mL significantly enhanced biofilm biomass by 1.82- to 4.27-fold and led to denser biofilm structures. Adhesion assays further revealed that fosfomycin significantly promoted the initial attachment of *S. aureus* to solid surfaces, a critical early step in biofilm development. Phenotypic analyses showed increased production of polysaccharide intercellular adhesin, enhanced bacterial aggregation, and accelerated autolysis, resulting in elevated extracellular DNA release. Enzymatic disruption experiments indicated that, in addition to PIA and eDNA, proteins also play an important role in fosfomycin-enhanced biofilm formation. RT-qPCR revealed significant upregulation of key biofilm-associated genes, including *icaA*, *icaB*, *fnbA*, *fnbB*, *emp*, *cidA*, and the global regulator *sarA*. Notably, deletion of *sarA* abolished fosfomycin-induced promotion of both adhesion and biofilm formation, while complementation restored the phenotype, confirming a *sarA*-dependent mechanism underlying the fosfomycin-mediated enhancement of biofilm formation. These findings suggest that sub-inhibitory concentrations of fosfomycin promote *S. aureus* biofilm formation via *sarA*-mediated regulation, involving increased PIA synthesis, eDNA release, and protein-dependent matrix components. This mechanism may contribute to treatment failure and the development of persistent, antibiotic-resistant infections.

**IMPORTANCE:**

Biofilm formation is a major factor in the persistence and antibiotic resistance of *Staphylococcus aureus* infections. Although fosfomycin is increasingly used to treat multidrug-resistant bacterial infections, its sub-inhibitory effects on biofilm formation have not been fully elucidated. Our study reveals that low-dose fosfomycin can significantly enhance *S. aureus* biofilm formation through a *sarA*-dependent mechanism. This finding raises concerns about the potential risks of sub-optimal dosing and highlights the need for careful evaluation of treatment strategies to avoid promoting persistent infections and resistance.

## INTRODUCTION

*Staphylococcus aureus* is a formidable pathogen responsible for a wide range of infections, from mild skin conditions to life-threatening diseases such as pneumonia, endocarditis, and sepsis (1). Its ability to form biofilms on both host tissues and medical devices further enhances its pathogenicity, complicating treatment due to increased antibiotic resistance and evasion of host immune responses (2, 3). This challenge is exacerbated by the propensity of *S. aureus* to develop resistance to multiple antibiotics, making it a significant concern in clinical treatment.

*S. aureus* biofilm formation is a dynamic process comprising three stages: attachment, maturation, and dispersion (4). The initial attachment phase involves bacterial adhesion to host surfaces through microbial surface components that recognize adhesive matrix molecules, such as fibronectin-binding proteins (FnbA and FnbB) and clumping factors (ClfA) (5). During maturation, cells proliferate and produce an extracellular matrix composed of polysaccharides, proteins, and extracellular DNA (eDNA) (6). A key component, polysaccharide intercellular adhesin (PIA), synthesized by the ica operon, facilitates bacterial adhesion and reinforces the biofilm structure (7). The ica operon includes genes *icaADBC*, which are essential for the synthesis and modification of PIA, with *icaA*-encoding N-acetylglucosaminyltransferase that initiates PIA synthesis and *icaB* responsible for the deacetylation of PIA to enhance its biofilm-forming capacity (7). Inhibiting PIA synthesis via the ica-dependent pathway is a well-established mechanism for impeding *S. aureus* biofilm formation (8). In addition, staphylococcal accessory regulator (SarA) binds with high affinity to the promoter region of *icaA*, regulating its expression (9). Apart from PIA, eDNA is another crucial component of the *S. aureus* biofilm matrix, released through autolysis (10). The Cid/Lrg holin-antiholin system regulates cell lysis and eDNA release during planktonic growth. In the dispersion phase, cells detach from the mature biofilm and colonize new sites, leading to persistent infections (11). Among the protein components of the biofilm matrix, the extracellular matrix protein (Emp) also plays an important role in stabilizing the biofilm structure and facilitating cell-cell interactions (12).

Fosfomycin, notable for its broad antibacterial spectrum and unique mechanism of action (13), penetrates bacterial cells primarily through two membrane transport systems: the l-α-glycerol-3-phosphate transporter (GlpT) and the glucose-6-phosphate (G-6-P) transporter (UhpT) (14). The UhpT system, induced by G-6-P, serves as an alternative to the GlpT system (15). Once inside the cell, fosfomycin covalently binds to the cysteine residue in the active site of the UDP-N-acetylglucosamine enolpyruvyl transferase (MurA), inhibiting bacterial cell wall synthesis (16). This action disrupts the integrity of the peptidoglycan layer, leading to cell lysis. Importantly, MurA is absent in humans and shares little homology with human proteins, making it an ideal target for therapeutic intervention (17). Fosfomycin’s clinical use has surged since 2010, when it was recommended as a first-line therapy for uncomplicated urinary tract infections (18). However, a recent study from China reported that the predominant *S. aureus* lineage ST5 in the Chinese region exhibited an alarming increase in fosfomycin resistance rates, rising from 19.5% in 2008 to 67.3% in 2019, with the majority of fosfomycin-resistant ST5 isolates (92.7%) displaying high-level resistance (19).

Sub-inhibitory concentrations of various antibiotics have been demonstrated to modulate MRSA biofilm formation (20). β-lactams (methicillin, ampicillin, and amoxicillin), mupirocin, and rifampin significantly enhance biofilm formation by influencing adhesion-related genes (*fnbA*, *fnbB*, *clfA*, *clfB*, and *icaA*) and autolysis-related genes (*atl* and *cidA*) (21–24). By contrast, sub-inhibitory concentrations of fusidic acid reduce *S. aureus* biofilm formation and suppress α-toxin expression (25). Investigating the effects of sub-inhibitory antibiotic concentrations on biofilm formation is crucial for developing specific therapeutic strategies for biofilm-associated *S. aureus* infections, optimizing antibiotic treatment regimens, and addressing the challenges posed by persistent and recurrent infections.

This study investigates the effects of sub-inhibitory concentrations of fosfomycin on *S. aureus* biofilm formation and elucidates the underlying mechanisms. By exploring how fosfomycin influences biofilm development through gene expression changes and phenotypic adaptations, this research provides valuable insights into optimizing antibiotic use to enhance treatment efficacy against device-associated and recurrent *S. aureus* infections.

## MATERIALS AND METHODS

### Bacterial strains, culture conditions, and chemicals

Detailed information on all *S. aureus* strains used in this study is provided in Table 1. *S. aureus* strains Newman and N315 were preserved in our laboratory. To investigate whether the biofilm enhancement effect of fosfomycin on *S. aureus* is universal, we selected clinical isolates with moderate biofilm-forming ability (0.2 ≤ OD_600_ <1.0) from the Clinical Microbiology Laboratory of the First Affiliated Hospital of Nanchang University, China. This selection criterion ensured that the isolates exhibited measurable levels of biofilm formation, allowing for the observation of potential changes in biofilm formation, whether increases or decreases, in response to fosfomycin treatment. Each isolate was identified using the MALDI-TOF mass spectrometry system (Bruker Daltonics, Billerica, MA, USA). In addition, the clinical isolates were typed by amplifying the internal fragments of seven housekeeping genes (arcC, aroE, glpF, gmk, pta, tpi, and yqiL) of *S. aureus*, following previously described methods (26). This approach allowed us to include a diverse set of strains with varying genetic backgrounds. Unless otherwise noted, *S. aureus* strains were cultured in Tryptic Soy Broth containing 0.5% glucose (TSBG) at 37°C with shaking at 220 rpm. Fosfomycin (cat. no: HY-B1075A) was obtained from MedChemExpress (Shanghai, China) and dissolved in sterile deionized water.

**TABLE 1:** Characteristics of laboratory and clinical *S. aureus* strains used in this study^*a*^

Bacterial strains	MSSA/MRSA	Source	MIC of FOS (μg/mL)	MLST
Newman	MSSA	NA^*b*^	4	ST254
N315	MRSA	NA^*b*^	4	ST5
MSSA23	MSSA	Blood	64	ST5
MSSA28	MSSA	Sputum	4	ST59
MRSA3	MRSA	Pus	32	ST239
MRSA7	MRSA	Sputum	128	ST5
MRSA14	MRSA	Blood	4	ST764
MRSA19	MRSA	Blood	4	ST59
MRSA24	MRSA	Sputum	4	ST7

^*a*^ MSSA, methicillin-sensitive *Staphylococcus aureus*; MRSA, methicillin-resistant *Staphylococcus aureus*; MIC, minimum inhibitory concentration; FOS, fosfomycin; MLST, multilocus-sequence typing.

^*b*^ NA, not applicable.

### Determining the minimum inhibitory concentration

The minimum inhibitory concentration (MIC) of oxacillin was determined using the broth microdilution method in accordance with the Clinical and Laboratory Standards Institute (CLSI, 2024) guidelines. Briefly, bacterial suspensions were prepared to match a 0.5 McFarland standard and subsequently diluted 1:100 in freshly prepared cation-adjusted Mueller-Hinton broth supplemented with 2% NaCl. The final bacterial inoculum was incubated at 35°C for 24 hours. Oxacillin was tested at concentrations ranging from 0.25 to 128 mg/L. Isolates with a MIC of ≥4 mg/L were interpreted as methicillin-resistant *Staphylococcus aureus* (MRSA). The MIC of fosfomycin was determined using the agar dilution method. According to EUCAST guidelines (https://www.eucast.org/clinical_breakpoints/), Mueller-Hinton agar plates were supplemented with 25 mg/L glucose-6-phosphate (G-6-P). Fosfomycin MICs were interpreted based on EUCAST clinical breakpoints, with resistance defined as a MIC >32 mg/L. *S. aureus* ATCC 29213 was used as the control strain. This experiment was performed in triplicate.

### Adhesion assay

The adhesion assay was conducted with slight modifications as previously described (27). Overnight cultures of *S. aureus* were diluted 1:200 in TSBG, with or without fosfomycin, and inoculated into 12-well plates (Corning, NY, USA). The same brand of plates was used for all experiments, regardless of well format. After static incubation at 37°C for for either 30 minutes or 2 hours, non-adherent cells were removed, and the wells were gently washed three times with PBS. To harvest the biofilms, adherent cells were thoroughly scraped off the well surfaces using a sterile cell scraper. The collected cells were then suspended in 1 mL sterile PBS. To minimize clumping and achieve a single-cell suspension, the bacterial cells underwent sonication with six 3 second pulses at 6 second intervals at 4°C, using a Vibra-Cell Sonicator set at a 40% duty cycle. The resulting suspensions were serially diluted for colony counting. Each experimental condition was tested with three technical replicates (three parallel wells per condition), and the entire experiment was independently repeated three times (biological replicates).

### Crystal violet staining assay

Biofilm formation was assessed using 96-well plates (Corning, NY, USA). Overnight cultures of *S. aureus* were diluted 1:200 in TSBG, with fosfomycin added to the bacterial suspension. A 200 µL TSBG suspension without fosfomycin served as the negative control. After incubating at 37°C for 24 hours, excess medium was removed, and the adhered biofilms were washed twice with sterile phosphate-buffered saline (PBS). The biofilms were then fixed with 200 µL of methanol for 15 minutes. After removing the methanol and air-drying, the biofilms were stained with 100 µL of 0.1% (wt/vol) crystal violet solution at room temperature for 15 minutes. Following staining, the wells were washed twice with sterile PBS. After staining and washing, the crystal violet was dissolved in the same well with the biofilm using 200 µL of 30% (vol/vol) acetic acid. The OD_600_ was measured directly from these wells without further dilution using a microplate reader. Each condition was tested using three technical replicates, and the entire experiment was independently repeated at least three times (biological replicates).

### Confocal laser scanning microscopy

*S. aureus* strains were cultured in confocal dishes (NEST Biotechnology, China) with TSBG containing 1 µg/ml fosfomycin. Confocal dishes without fosfomycin served as controls. After 24 hours of incubation at 37°C, the dishes were washed twice with PBS. The biofilms were then stained with SYTO9 (Invitrogen, Thermo Fisher Scientific, USA) and propidium iodide (PI) reagents (Invitrogen, Thermo Fisher Scientific, USA) at concentrations of 3 µM and 20 µM, respectively. The samples were incubated in the dark for 30 minutes. The stained biofilm samples were observed using confocal laser scanning microscopy (CLSM; Leica).

### Immunoblotting for PIA

Overnight cultures of *S. aureus* were diluted 1:200 in TSBG, with or without fosfomycin (final concentration 1 µg/mL), and incubated in six-well plates (Corning, NY, USA) at 37°C for 24 hours. The wells were then washed three times with PBS to remove planktonic bacteria. The surface-adhered bacteria (biofilm) were scraped and collected. Equal amounts of bacteria were obtained by centrifugation. The bacteria were boiled for 10 minutes and centrifuged for 10 minutes. Forty microliter of the supernatant from each sample was mixed with 20 µL of proteinase K (20 mg/mL). Ten microliter of the mixture was spotted onto a nitrocellulose membrane. The membrane was dried and blocked overnight at 4°C with 5% BSA (bovine serum albumin, Biosharp, Beijing, China). After washing with phosphate-buffered saline containing 0.1% Tween-20 (PBST), the membrane was incubated with HRP (horseradish peroxidase)-conjugated wheat germ agglutinin (1/2,500 dilution) at 37°C for 1 hour. The membrane was washed three times with PBST (10 minutes each) and visualized using enhanced chemiluminescence (ECL Plus; Beyotime). This experiment was performed in triplicate.

### Cell aggregation assay

Cell aggregation was analyzed as previously described (28). Briefly, *S. aureus* was diluted 1:200 into 2 mL TSBG, with or without fosfomycin (final concentration 1 µg/mL). Cultures were incubated at 37°C and 220 rpm for 24 hours; untreated samples served as controls. Cells were collected by centrifugation at 5,000 × *g* for 10 minutes, washed twice with PBS, and resuspended in 3 mL PBS. The OD_600_ was adjusted to 1.5 (initial OD) in clean glass tubes and left to stand at room temperature for 24 hours. The supernatant was aspirated, and cells were resuspended in 3 mL PBS. The OD_600_ (final OD) was measured by carefully removing a 100 µL aliquot from the top of the tube. The percentage of cell aggregation was calculated as (initial OD − final OD)/initial OD ×100%. The relative aggregation of fosfomycin-treated samples was expressed as a percentage of the untreated control (100%). This experiment was performed in triplicate.

### Triton X-100-induced autolysis

The autolysis assay was performed according to previously established methods (28). Overnight cultures of *S. aureus* were diluted 1:200 into 50 mL TSB containing 1 M NaCl. When the culture reached an OD_600_ of 0.2, an aliquot was treated with 1 µg/mL fosfomycin. Incubation continued until the OD_600_ reached 0.6–0.8. The cultures were then centrifuged at 4°C and 4,000 × *g* for 20 minutes. The resulting bacterial pellets were gently washed with pre-cooled deionized water. Bacteria were resuspended in lysis buffer containing 0.05% Triton X-100 and 50 mM Tris-Cl (pH 7.2), and the OD_600_ was adjusted to 1.0. The suspensions were incubated at 37°C and 220 rpm for 3 hours, with OD_600_ values recorded every hour. This experiment was performed in triplicate to ensure accuracy.

### Quantitative analysis of eDNA

The isolation and quantification of eDNA were performed as previously described (23). *S. aureus* strains were cultured in six-well plates (Corning, NY, USA) as described earlier. Biofilms were resuspended in freshly prepared TES buffer (50 mM Tris-HCl, pH 8.0; 10 mM EDTA, 500 mM NaCl). Samples were centrifuged at 14,000 rpm for 5 minutes, and the supernatants were transferred to new tubes. Equal volumes of phenol-chloroform-isoamyl alcohol (25:24:1) and chloroform-isoamyl alcohol (24:1) were added to the supernatants. The tubes were mixed thoroughly and stored overnight at −20°C with the addition of 10% 3M sodium acetate in ethanol. eDNA was collected by centrifugation at 16,000 rpm for 20 minutes, washed with 75% ethanol, and dissolved in TE buffer. eDNA quantification was performed using a NanoDrop 2000 spectrophotometer. To account for potential differences in biomass, the average OD_600_ of each unscraped biofilm was determined. The relative amount of eDNA secretion was determined by dividing the total eDNA (ng) by the OD_600_ of the biofilm. This experiment was performed in triplicate.

### Proteinase K biofilm degradation assay

To investigate the contribution of protein components to the structural integrity of fosfomycin-induced biofilms, a proteinase K degradation assay was performed. *S. aureus* cultures were grown in 96-well polystyrene microtiter plates (Corning, NY, USA) with TSBG and 1/4 MIC of fosfomycin for 24 hours at 37°C. After incubation, the culture medium was gently aspirated, and each well was washed twice with sterile PBS to remove non-adherent cells. Subsequently, 200 µL of TSBG containing various concentrations of proteinase K was added to each well. Control wells received an equivalent volume of TSBG with PBS but without proteinase K. The plates were then incubated statically at 37°C for an additional 4 hours to allow enzymatic degradation of protein components within the biofilm matrix. After treatment, the wells were washed twice with PBS to remove residual enzyme and any detached cells. The remaining adherent biofilms were fixed with methanol, stained with 0.1% (wt/vol) crystal violet, and then dissolved in acetic acid. The biofilm biomass was quantified by measuring OD_600_ as described above. Each experimental condition was tested in triplicate, and all experiments were independently repeated at least three times.

### Real-time quantitative PCR

*S. aureus* strains were cultured in TSBG at 37°C for 24 hours, with or without 1 µg/mL fosfomycin. RNA extraction was performed using the Total RNA Purification Kit (Sangon Biotech). Reverse transcription was carried out using the PrimeScript RT Reagent Kit with gDNA Eraser (Takara). Quantitative PCR analysis was conducted using an ABI 7500 detection system with TB Green Premix Ex Taq (Takara). The relative changes in RNA expression of target genes were calculated using the 2^−ΔΔCt^ method (29). Briefly, the ΔCt was calculated as the difference between the Ct values of the target gene and the gyrB gene (used as the reference gene). The ΔΔCt was then calculated as the difference between the ΔCt of the fosfomycin-treated samples and the ΔCt of the untreated control samples. The relative expression of the target genes was determined as 2^−ΔΔCt^. The primers used are listed in Table 2. For each sample, qPCR was performed in triplicate wells (technical replicates), and the entire experiment was independently repeated at least three times (biological replicates).

**TABLE 2:** PCR primers are used in this study

PCR product	Primer description	Primer sequence
*gyrB*-RT-F	qPCR	ACATTACAGCAGCGTATTAG
*gyrB*-RT-R	qPCR	CTCATAGTGATAGGAGTCTTCT
*icaA*-RT-F	qPCR	TCAGATAATACAGCAGAACTCAT
*icaA*-RT-R	qPCR	GCATCCAAGCACATTACATAA
*clfA*-RT-F	qPCR	GCTTCAGTGCTTGTAGGTA
*clfA*-RT-R	qPCR	GCTATCAGATTGCGTAACAC
*fnbA*-RT-F	qPCR	TTCCTTAACTACCTCTTCT
*fnbA*-RT-R	qPCR	CAATCATATAACGCAACAG
*atl*-RT-F	qPCR	GGCTTAGGTGTTGGTGTA
*atl*-RT-R	qPCR	TATGGCTCTGTGAATGGTAA
*cidA*-RT-F	qPCR	TGTACCGCTAACTTGGGTAGAAGAC
*cidA*-RT-R	qPCR	CGGAAGCAACATCCATAATACCTAC
*sarA*-RT-F	qPCR	TTCGCTGATGTATGTCAAT
*sarA*-RT-R	qPCR	GAGTTGTTATCAATGGTCAC
*sarA*-U-F	Knockout	GGGGACAAGTTTGTACAAAAAAGCAGGCTATGAGGCACATATTCTAGGA
*sarA*-U-R	Knockout	ATCAAATAGGGAGGTTTTAAACGAATCACTGAAGCAAACAAC
*sarA*-D-F	Knockout	GTTGTTTGCTTCAGTGATTCGTTTAAAACCTCCCTATTTGAT
*sarA*-D-R	Knockout	GGGGACCACTTTGTACAAGAAAGCTGGGTTCGATAGTCAACTCATTCTT
*sarA*-com-F	Complementation	GGATCCAAGAGTTAAGCTATAACAAAGA
*sarA*-com-F	Complementation	AAGCTTTTATAGTTCAATTTCGTTGTTT

### Construction of gene deletion mutants and complementation mutants

Gene deletion mutants of the *S. aureus* Newman strain were constructed using the pKOR1 plasmid for homologous recombination, as previously described by Bae and Schneewind et al., with minor modifications (30). Briefly, the upstream and downstream DNA fragments (1,000 bp each) of the *sarA* gene were amplified from Newman chromosomal DNA. These fragments were fused by overlap extension PCR and cloned into the pKOR1 vector using the Gateway BP Clonase II enzyme (Thermo Fisher Scientific), generating the recombinant plasmid pKOR1 Δ*sarA*. The resulting plasmids were first transformed into *E. coli* DH5α and DC10B strains and subsequently electroporated into the Newman strain. Homologous recombination between the plasmid and the genomic arms, induced by chloramphenicol selection and incubation at 42°C, resulted in the deletion of *sarA*. Subsequently, plasmid elimination was performed. Briefly, pKOR1, a temperature-sensitive shuttle plasmid, remains stable at 30°C but is prone to loss at 42°C in *S. aureus*. Bacteria that had undergone homologous recombination were subjected to multiple passages at elevated temperatures to promote plasmid loss. The bacteria were then plated simultaneously on agar plates with and without chloramphenicol. Putative gene mutants were identified as those strains that failed to grow in the presence of chloramphenicol but thrived in antibiotic-free conditions. This approach allowed for the elimination of the plasmid and isolation of the desired *sarA* deletion mutant. The successful deletion of the genes was confirmed by conducting a PCR with primers designed to bind to sequences upstream and downstream of the deleted region. This PCR amplified a shorter fragment compared to the wild type only if the gene deletion was successful. Following this, sequencing was performed on the PCR products to provide further confirmation of the gene deletion. For the complementation of the *sarA* deletion mutant, the full-length *sarA* gene along with its native promoter region was PCR-amplified and ligated into the pLi50 plasmid using T4 DNA ligase. The resulting complementation plasmid, pLi50-*sarA*, was then electroporated into the *sarA* deletion mutant (Δ*sarA*), resulting in the generation of the complemented strain (Δ*sarA*-C). Primers used for these constructions are listed in Table 2.

### Statistical analysis

Data analyses were conducted using GraphPad Prism 9.0 software. Statistical significance was analyzed with one-way analysis of variance (ANOVA) or unpaired two-tailed t-tests, with a *P*-value less than 0.05 considered statistically significant. Error bars on the graphs represent the standard deviation (mean ± SD). Significance is indicated as follows: *P < 0.05, **P < 0.01, ***P < 0.001, ****P < 0.0001, ns (not significant) *P* > 0.05.

## RESULTS

### Sub-inhibitory concentrations of fosfomycin enhanced the adhesion ability of *S. aureus*

**Fig 1:**
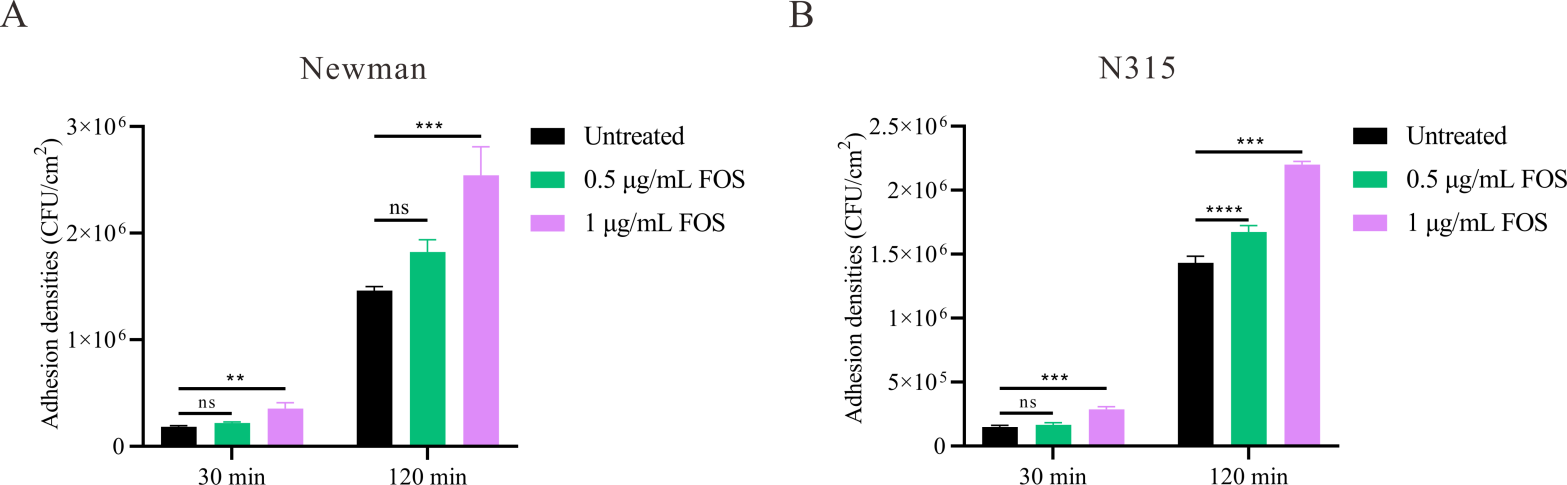
Effect of sub-inhibitory concentrations of fosfomycin on the initial adhesion ability of *S. aureus*. (**A**) Adhesion densities of *S. aureus* Newman strain at 30 and 120 minutes under different fosfomycin concentrations (0, 0.5, and 1 µg/mL). (**B**) Adhesion densities of *S. aureus* N315 strain under the same conditions. Adhesion is quantified as colony-forming units (CFU) per cm². Data are presented as mean ± SEM. Statistical analysis was performed using one-way ANOVA. **P < 0.01, ***P < 0.001, ****P < 0.0001, ns (not significant) *P* > 0.05.

The MLST typing revealed that Newman and N315 strains are classified as ST254 and ST5, respectively. The agar dilution method determined that the MIC of fosfomycin for *S. aureus* Newman (MSSA) and N315 (MRSA) was 4 µg/mL. We investigated the effect of sub-inhibitory concentrations (0.5 µg/mL and 1 µg/mL) of fosfomycin on the initial adhesion phase of biofilm formation for these two representative laboratory strains. The adhesion assay results indicated that, compared to the control group, fosfomycin at a concentration of 1 µg/mL significantly enhanced the adhesion to solid surfaces for both the Newman and N315 strains (Fig. 1). For the Newman strain (Fig. 1A), at 30 minutes, there was no significant difference in adhesion between untreated cells and those treated with 0.5 µg/mL fosfomycin. However, treatment with 1 µg/mL fosfomycin resulted in a significant increase in adhesion (*P* = 0.0001). At 120 minutes, both concentrations of fosfomycin significantly enhanced adhesion compared to untreated cells. The adhesion density increased from an average of (1.43 ± 0.05) ×10^6^ CFU/cm^2^ in untreated cells to (1.68 ± 0.05) ×10^6^ CFU/cm^2^ with 0.5 µg/mL fosfomycin (*P* < 0.0001), and further to (2.20 ± 0.03) ×10^6^ CFU/cm^2^ with 1 µg/mL fosfomycin (*P* = 0.001). The N315 strain (Fig. 1B) exhibited a similar trend, albeit with some differences. At 30 minutes, only the 1 µg/mL fosfomycin treatment showed a significant increase in adhesion (*P* = 0.0013). At 120 minutes, while the 0.5 µg/mL treatment did not produce a statistically significant change, increasing the adhesion density from (1.46 ± 0.04) ×10^6^ CFU/cm^2^ in untreated cells to (1.82 ± 0.12) ×10^6^ CFU/cm^2^, the 1 µg/mL treatment significantly enhanced adhesion to (2.54 ± 0.27) ×10^6^ CFU/cm^2^ (*P* = 0.0004). These findings suggest that sub-inhibitory concentrations of fosfomycin can significantly enhance the adhesion ability of *S. aureus*.

### Sub-inhibitory concentrations of fosfomycin enhanced biofilm formation ability of *S. aureus*

**Fig 2:**
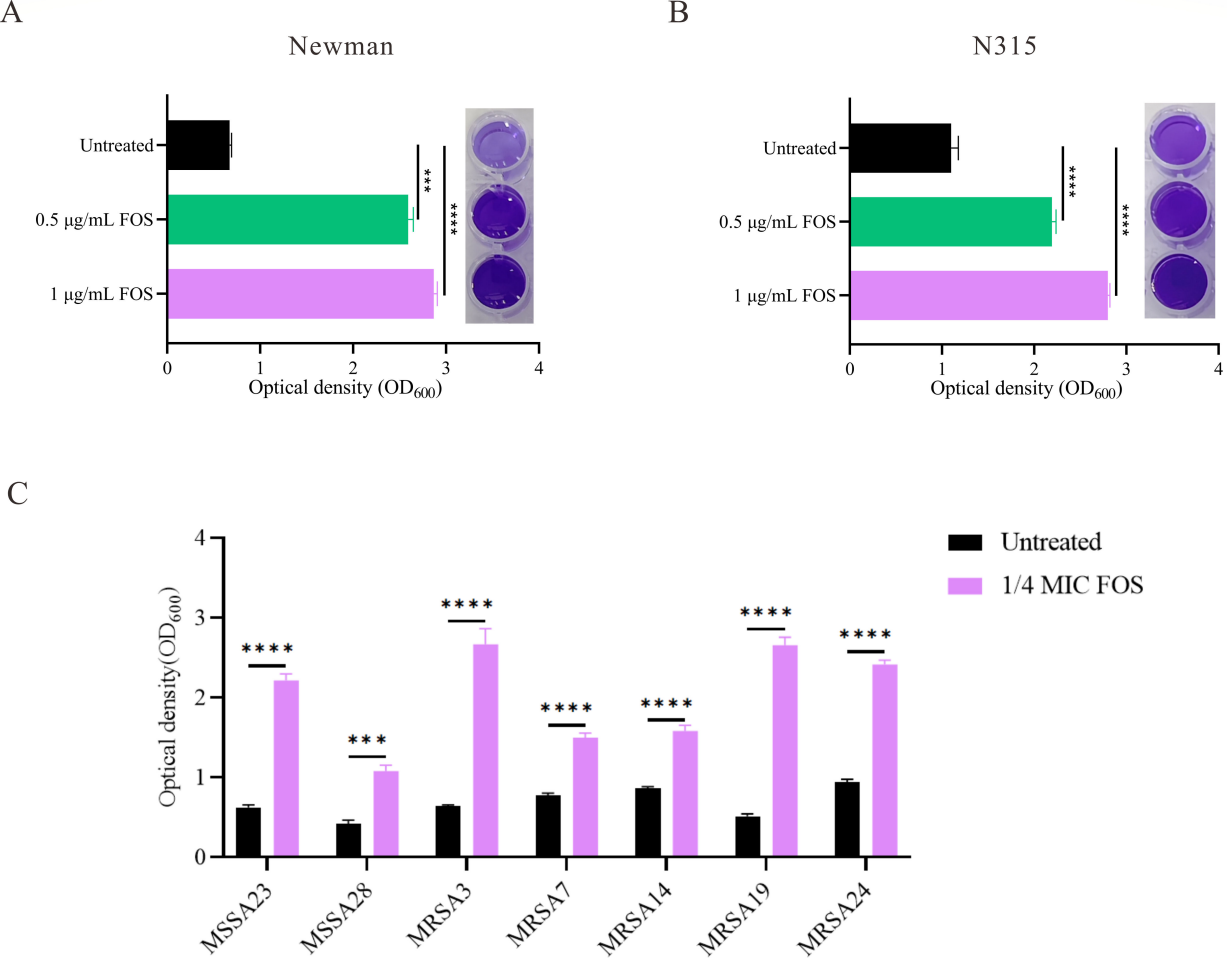
Effect of sub-inhibitory concentrations of fosfomycin on biofilm formation ability of *S. aureus*. Crystal violet staining and quantification of biofilms formed by *S. aureus* Newman (**A**) and N315 (**B**) strains under sub-inhibitory concentrations of fosfomycin on the biofilm formation ability of *S. aureus*. Statistical analysis was performed using one-way ANOVA. (**C**) Effect of fosfomycin (1/4 MIC) on biofilm formation of various clinical *S. aureus* isolates. The concentration of fosfomycin at 1/4 MIC was calculated based on the MIC value determined for each strain, as listed in Table 1. Data are presented as mean ± SEM. Statistical analysis was performed using an unpaired, two-tailed Student’s t-test. ***P < 0.001, ****P < 0.0001.

The crystal violet staining method was employed to evaluate the effect of fosfomycin on biofilm formation by the Newman and N315 strains. As shown in Fig. 2A, exposure to fosfomycin led to a visually observable dose-dependent increase in biofilm biomass for both strains. Quantitative analysis revealed that for the Newman strain, the optical density (OD_600_) increased from 0.671 ± 0.021 in untreated cells to 2.593 ± 0.056 at 0.5 µg/mL fosfomycin, representing a 3.86-fold increase (*P =* 0001). At 1 µg/mL fosfomycin, the OD_600_ further increased to 2.869 ± 0.037, indicating a 4.27-fold increase compared to untreated cells (*P <* 0.0001). For the N315 strain (Fig. 2B), the biofilm biomass increased from an OD_600_ of 1.100 ± 0.075 in untreated cells to 2.191 ± 0.045 at 0.5 µg/mL fosfomycin (1.99-fold increase), and further to 2.799 ± 0.021 at 1 µg/mL fosfomycin (2.55-fold increase) (*P <* 0.0001 for both concentrations). To investigate whether the enhancement of biofilm formation by fosfomycin is a general phenomenon, we conducted crystal violet staining assays on seven clinical isolates of *S. aureus* with moderate biofilm-forming ability (0.2 ≤ OD_600_ <1.0). These isolates included 2 MSSA and 5 MRSA strains, detailed in Table 1. Among the seven clinical isolates, two strains (MSSA23 and MRSA7) exhibited fosfomycin resistance (MICs of 64 µg/mL and 128 µg/mL, respectively). Notably, both resistant strains belonged to the ST5 lineage, while all non-ST5 isolates were sensitive to fosfomycin (MIC ≤32 µg/mL). The effect of fosfomycin on biofilm formation of these clinical isolates is shown in Fig. 2C. Overall, sub-inhibitory concentrations of fosfomycin (1/4 MIC) induced a consistent increase in *S. aureus* biofilm biomass, ranging from 1.82- to 4.27-fold compared to the untreated controls.

**Fig 3:**
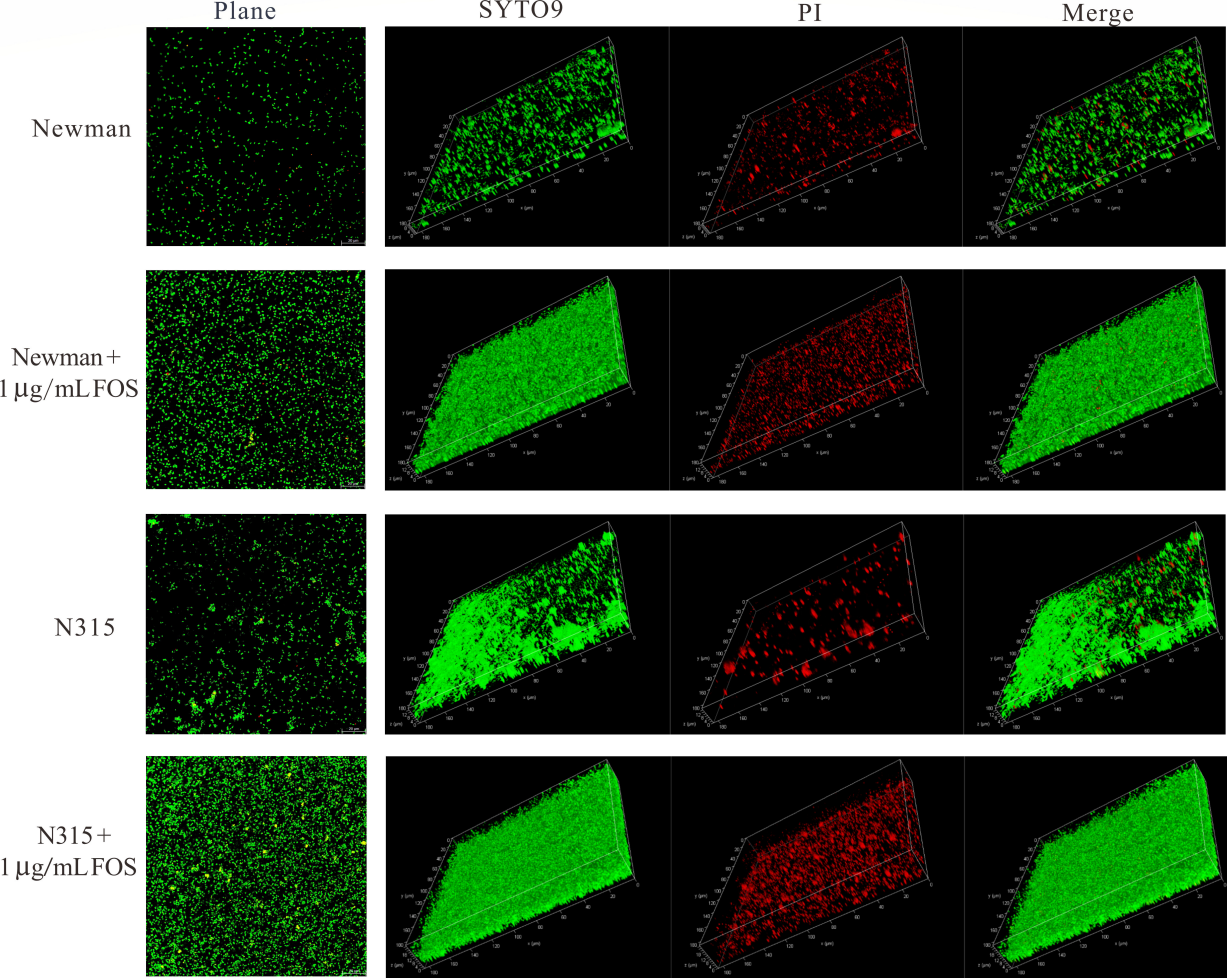
Confocal laser scanning microscopy analysis of *S. aureus* biofilm formation under sub-inhibitory concentrations of fosfomycin. Representative images of biofilms formed by *S. aureus* Newman and N315 strains with and without 1 µg/mL fosfomycin treatment. Bacteria were stained with SYTO9 (green, live cells) and propidium iodide (PI, red, dead cells). Images show magnification, SYTO9 channel, PI channel, and merged views for each condition.

To further elucidate the biofilm-enhancing effect of fosfomycin, CLSM was employed to analyze biofilm architecture in the *S. aureus* Newman and N315 strains. The biofilms were visualized using SYTO9/PI staining to differentiate between live (green) and dead (red) cells. As shown in Fig. 3, untreated Newman and N315 strains formed relatively sparse biofilms, characterized by scattered clusters of viable cells with limited structural complexity. By contrast, treatment with sub-inhibitory concentrations of fosfomycin (1 µg/mL) notably increased biofilm density in both strains. In the fosfomycin-treated Newman strain, CLSM revealed a more compact and homogeneous biofilm structure with a substantial increase in the number of viable cells, as demonstrated by the intensified green fluorescence. The treated biofilm also displayed a thicker three-dimensional structure, suggesting enhanced biofilm maturation. Similarly, the N315 strain exhibited a significant increase in biofilm biomass and cell viability following fosfomycin exposure. The treated N315 biofilms appeared denser and more uniformly distributed across the surface, with fewer dead cells (as indicated by the diminished red fluorescence), pointing to a more robust biofilm formation process. These observations consistently demonstrate that sub-inhibitory concentrations of fosfomycin promote the formation of denser and more viable biofilms in both the Newman and N315 strains.

### Sub-inhibitory concentrations of fosfomycin promoted PIA synthesis, eDNA release, and protein-dependent Biofilm Formation in *S. aureus*

**Fig 4:**
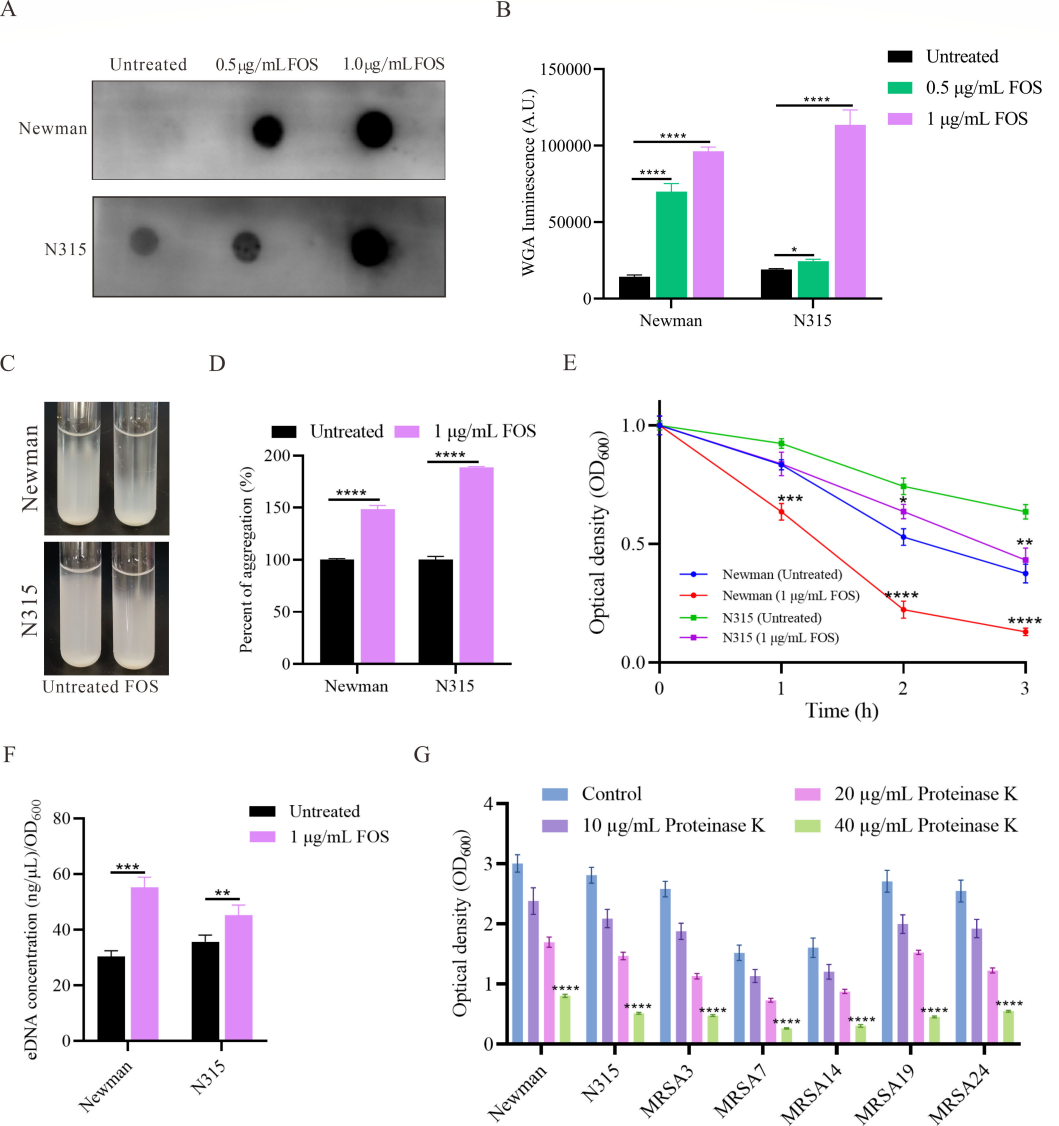
Effect of sub-inhibitory concentrations of fosfomycin on major biofilm components of *S. aureus*. (**A**) Dot blot analysis of PIA production in Newman and N315 strains under different fosfomycin concentrations (0, 0.5, and 1 µg/mL). (**B**) Evaluation of gray values of PIA using ImageJ software. WGA: wheat germ agglutinin. Statistical analysis was performed using one-way ANOVA. (**C**) Visual assessment of *S. aureus* aggregation in Newman and N315 strains with and without 1 µg/mL fosfomycin treatment. (**D**) Quantitative analysis of *S. aureus* aggregation. (**E**) Autolysis assay of *S. aureus* Newman and N315 strains treated with or without 1 µg/mL fosfomycin. The autolysis rate is represented by the decrease in optical density (OD_600_) over time. Data points represent mean ± SEM. (**F**) Quantification of eDNA release in Newman and N315 strains with and without 1 µg/mL fosfomycin treatment. eDNA concentration is normalized to OD_600_ and expressed as ng/μL/OD_600_. (**G**) Quantification of biofilm biomass in Newman, N315, and five clinical MRSA isolates. Biofilms were first induced by 1/4 MIC fosfomycin for 24 hours, where the 1/4 MIC for each strain was calculated based on its individual MIC value, as shown in Table 1. After induction, biofilms were treated with different concentrations of proteinase K (10, 20, and 40 µg/mL) for 4 hours. Biofilm biomass was measured by crystal violet staining and expressed as OD_600_. Bars represent mean ± SEM. Statistical analysis was performed using one-way ANOVA. **P < 0.01, ***P < 0.001, ****P < 0.0001.

PIA, eDNA, and proteins are major components of the *S. aureus* biofilm matrix. To determine whether PIA is involved in fosfomycin-induced biofilm formation, we extracted and quantified PIA using the dot blot assay. The results showed a significant, dose-dependent increase in PIA levels in the fosfomycin-treated Newman and N315 strains compared to the control group (Fig. 4A and B). This indicates that fosfomycin promotes PIA synthesis, leading to the formation of a biofilm matrix rich in PIA. Enhanced PIA production can increase the self-aggregation of *S. aureus*. Therefore, we incubated the *S. aureus* strains under static conditions and observed the degree of aggregation after 24 hours (Fig. 4C). For the Newman strain, treatment with 1 µg/mL fosfomycin significantly increased the aggregation rate to 188.67 ± 0.83% compared to the untreated control (100% ± 3.20%), representing a 1.89-fold increase (*P* < 0.0001, Fig. 4D). Similarly, the N315 strain exhibited a significant enhancement in aggregation when exposed to 1 µg/mL fosfomycin, with the aggregation rate increasing to 148.67% ± 3.51% compared to the untreated control (100% ± 1.20%), indicating a 1.49-fold increase (*P* < 0.0001).

Autolysis of *S. aureus* can release eDNA, which facilitates biofilm formation. To investigate the effect of fosfomycin on eDNA, another important biofilm component, we conducted Triton X-100 autolysis assays. Compared to the control group, the fosfomycin-treated Newman strain exhibited significantly accelerated autolysis kinetics from 1 to 3 hours, and the N315 strain showed similar accelerated autolysis kinetics from 2 to 3 hours (Fig. 4E). Quantification of eDNA revealed a significant increase in eDNA release in both Newman and N315 strains when treated with sub-inhibitory concentrations of fosfomycin (Fig. 4F). For the Newman strain, eDNA concentration increased from 30.37 ± 2.07 ng/µL/OD_600_ in untreated cells to 55.29 ± 3.57 ng/µL/OD_600_ in cells treated with 1 µg/mL fosfomycin, representing a 1.82-fold increase (*P* = 0.0005). Similarly, the N315 strain showed an increase in eDNA concentration from 35.70 ± 2.35 ng/µL/OD_600_ in untreated cells to 45.29 ± 3.56 ng/µL/OD_600_ in fosfomycin-treated cells, indicating a 1.27-fold increase (*P* = 0.018). These results indicate that sub-inhibitory concentrations of fosfomycin enhance PIA production and eDNA release, thereby promoting biofilm formation of *S. aureus*.

To elucidate the contribution of protein components to fosfomycin-enhanced biofilm formation, mature biofilms induced by sub-inhibitory concentrations of fosfomycin (1/4 MIC) were treated with proteinase K at final concentrations of 10, 20, and 40 µg/mL for 4 hours. These biofilms were formed by *S. aureus* Newman and N315 strains, as well as five clinical MRSA isolates in Table 1. As shown in Fig. 4G, treatment with proteinase K resulted in a significant, dose-dependent reduction in biofilm biomass. At the concentration of 40 µg/mL, biofilm biomass was reduced by 73.2% to 83.5% compared to the untreated control group. These results indicate that, in addition to PIA and eDNA, proteins also play a significant role in fosfomycin-enhanced biofilm formation.

### Effect of sub-inhibitory concentrations of fosfomycin on the expression of biofilm-related genes

**Fig 5:**
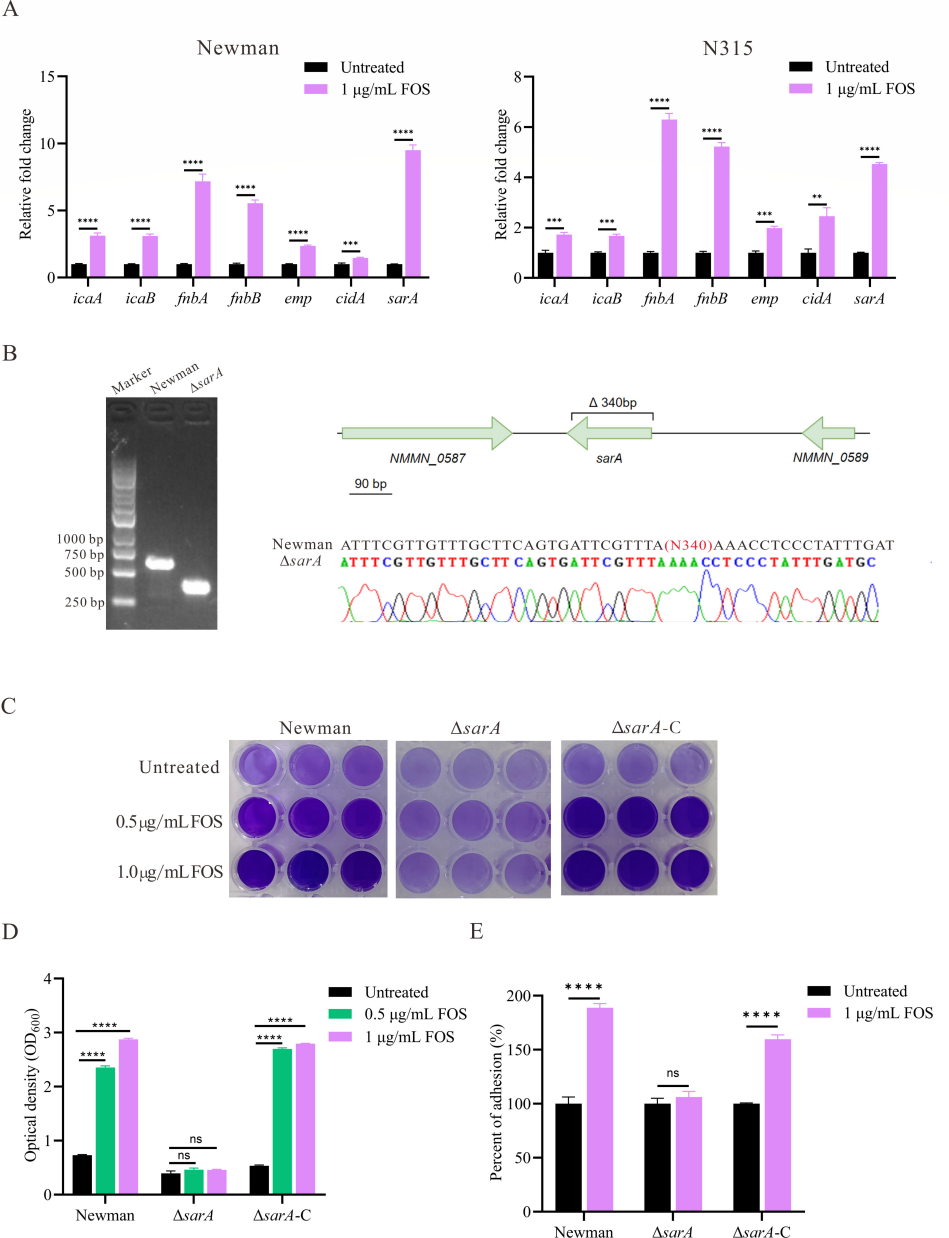
The mechanism by which sub-inhibitory concentrations of fosfomycin enhance *S. aureus* biofilm formation. (**A**) Effects of sub-inhibitory concentrations of fosfomycin on the expression of biofilm-related genes of *S. aureus*. Relative fold changes in gene expression in Newman (left) and N315 (right) strains treated with 1 µg/mL fosfomycin compared to untreated controls. Statistical analysis was performed using an unpaired, two-tailed Student’s t-test. (**B**) Confirmation of the *sarA* deletion (Δ*sarA*) in the Newman strain. Agarose gel electrophoresis (left; GL DNA Marker 1000, Accurate Biology) and Sanger sequencing results (right) are shown. (**C**) Crystal violet staining of biofilms formed by Newman and Δ*sarA*, Δ*sarA*-C (Δ*sarA* complemented with the sarA gene) strains under different fosfomycin concentrations (0, 0.5, and 1 µg/mL) in 96-well plates. (**D**) Quantification of biofilm biomass at OD_600_ after acetic acid dissolution. Data are presented as mean ± SEM. Statistical analysis was performed using one-way ANOVA. (**E**) Initial adhesion assays of Newman, Δ*sarA*, Δ*sarA*-C (Δ*sarA* complemented with the *sarA* gene) strains with and without 1 µg/mL fosfomycin treatment. Results are expressed as percentages of adhesion. Statistical analysis was performed using an unpaired, two-tailed Student’s t-test. **P < 0.01, ***P < 0.001, ****P < 0.0001, ns (not significant) *P* > 0.05.

To elucidate the mechanism by which fosfomycin enhances biofilm formation, we analyzed the mRNA transcript levels of biofilm-related genes in fosfomycin-treated and untreated *S. aureus* strains using RT-qPCR. As illustrated in Fig. 5A, treatment with 1 µg/mL fosfomycin significantly upregulated the expression of biofilm-related genes in both Newman and N315 strains. In the Newman strain, the most pronounced upregulation was observed in *sarA* (9.51 ± 0.38-fold, *P* < 0.0001) and fnbA (7.18 ± 0.54-fold, *P* < 0.0001). In addition, other genes exhibited substantial increases in expression: icaA (3.12 ± 0.20-fold, *P* < 0.0001), icaB (3.09 ± 0.15-fold, *P* < 0.0001), fnbB (5.54 ± 0.25-fold, *P* < 0.0001), emp (2.35 ± 0.07-fold, *P* < 0.0001), and *cidA* (1.46 ± 0.05-fold, *P* = 0.009). Although the N315 strain showed relatively lower upregulation compared to the Newman strain, significant increases were observed across all tested genes: *sarA* (4.53 ± 0.06-fold, *P* < 0.0001), fnbA (6.30 ± 0.24-fold, *P* < 0.0001), fnbB (5.23 ± 0.16-fold, *P* < 0.0001), emp (1.98 ± 0.07-fold, *P* = 0.0001), *cidA* (2.46 ± 0.34-fold, *P* = 0.002), icaA (1.72 ± 0.09-fold, *P* = 0.0008), and icaB (1.67 ± 0.06-fold, *P* = 0.0001). These findings corroborate our previous phenotypic analyses of biofilm formation, suggesting that fosfomycin enhances both *S. aureus* adhesion capabilities and the production of PIA and eDNA.

### Fosfomycin promotes biofilm formation of *S. aureus* in a *sarA*-dependent manner

Based on our previous gene expression analysis showing significant upregulation of *sarA*, and considering its role as a major transcriptional regulator of biofilm formation in *S. aureus*, we hypothesized that SarA might play a crucial role in fosfomycin-enhanced biofilm formation. To test this hypothesis, we generated a deletion mutant of *sarA* (Δ*sarA*) and its complemented strain (Δ*sarA*-C). PCR amplification of the regions flanking the *sarA* gene and agarose gel electrophoresis showed that the Δ*sarA* mutant had a band approximately 300 bp shorter than the Newman strain (Fig. 5B, left). Sanger sequencing further confirmed the successful deletion of *sarA* (Fig. 5B, right). Crystal violet staining revealed that the fosfomycin-induced enhancement of biofilm formation was markedly inhibited in the Δ*sarA* mutant even in the presence of 1 µg/mL fosfomycin (Fig. 5C). Importantly, complementation of sarA (Δ*sarA*-C) restored the fosfomycin-enhanced biofilm formation to levels comparable to those observed in the Newman strain (Fig. 5C and D). To further characterize the role of SarA in this process, we assessed the effect of fosfomycin on bacterial adhesion to solid surfaces, a critical initial step in biofilm formation. Fosfomycin treatment (1 µg/mL) failed to enhance the adhesion of the Δ*sarA* mutant to solid surfaces (*P* = 0.199). By contrast, both the Newman strain and the Δ*sarA*-C strain exhibited significantly enhanced adhesion upon fosfomycin treatment (*P* < 0.0001, Fig. 5E). These findings collectively demonstrate that *SarA* is essential for fosfomycin-enhanced biofilm formation in *S. aureus*, mediating both the initial adhesion process and subsequent biofilm development.

## DISCUSSION

Biofilm formation is associated with various types of persistent infections, as biofilm-encased bacteria can act as secondary invasive agents, leading to bloodstream infections (31, 32). In addition, the negatively charged exopolysaccharides within biofilms bind and limit the diffusion of antimicrobials, making the eradication of biofilm-embedded bacteria particularly challenging (33, 34). Fosfomycin, a phosphonic acid derivative, exhibits activity against a broad range of Gram-positive and Gram-negative pathogens. It is administered orally or intravenously for the treatment of uncomplicated urinary tract infections or severe infections (14).

Sub-inhibitory concentrations of antibiotics, arising from improper dosing or poor tissue penetration, have been implicated in inducing bacterial biofilm formation and exacerbating chronic infections (35, 36). Consequently, such sub-inhibitory drug concentrations may occur *in vivo*. Several studies have suggested that antibiotics like β-lactams can stimulate the expression of virulence factors in *S. aureus*, potentially impacting the prognosis of severe infections (20, 37). Importantly, for MRSA infections, sub-inhibitory drug concentrations not only lead to ineffective or failed treatment but may also enhance MRSA virulence and colonization, resulting in adverse outcomes (38). This underscores the clinical relevance and significance of the present study. In this study, we found that sub-inhibitory concentrations of fosfomycin significantly enhanced the initial adhesion ability of the laboratory strains Newman and N315. Crystal violet staining results demonstrated that sub-inhibitory fosfomycin concentrations significantly augmented biofilm formation in Newman, N315, and clinical *S. aureus* isolates with diverse genetic backgrounds. Notably, we observed considerable variation in fosfomycin MICs among the clinical isolates. Both fosfomycin-resistant strains (MSSA23 and MRSA7) were identified as ST5, which is consistent with previous reports from China indicating a high prevalence of fosfomycin resistance among ST5 *S. aureus* lineages (19). These findings suggest that fosfomycin resistance may be associated with specific clonal lineages, particularly ST5, which has been reported to harbor resistance determinants at a higher frequency (19, 39).

In the present study, we demonstrated that fosfomycin-induced *S. aureus* biofilm formation is associated with enhanced PIA production, eDNA release, and adhesive surface proteins. RT-qPCR analysis showed upregulation of genes related to PIA synthesis (icaA and icaB), autolysis and eDNA release (cidA), fibronectin-binding proteins (fnbA and fnbB), the extracellular matrix protein (emp), and the key regulator *sarA*, providing a molecular basis for the observed biofilm enhancement. Functional analysis using a *sarA* knockout mutant further confirmed that fosfomycin-induced biofilm formation is primarily mediated through *SarA* regulation of the ica operon. Although this mechanism shares certain similarities with those reported for other antibiotics, it also exhibits important distinctions. Previous studies have shown that sub-inhibitory concentrations of mupirocin and clindamycin often enhance biofilm formation via increased eDNA release and alternative regulatory pathways such as cidA-mediated autolysis or σB-dependent stress responses (24, 40). By contrast, our results indicate that fosfomycin-induced biofilm is predominantly dependent on PIA and proteins. Although fosfomycin treatment also increased eDNA production, the loss of biofilm enhancement in the Δ*sarA* mutant suggests that eDNA plays a limited structural role in this context. We hypothesize that fosfomycin-induced eDNA release in *S. aureus* may be related to its bactericidal mechanism, which disrupts cell wall synthesis by inhibiting MurA and leads to bacterial lysis, but that this eDNA does not substantially contribute to the biofilm matrix. Collectively, these findings highlight the antibiotic-specific nature of biofilm adaptation in *S. aureus*, where different antibiotics activate distinct regulatory networks and matrix compositions. Our study emphasizes the central role of *SarA*-mediated PIA synthesis in the response to sub-inhibitory concentrations of fosfomycin, distinguishing it from the mechanisms underlying biofilm enhancement by other antibiotics. This outcome underscores the complexity of *S. aureus* biofilm regulation and suggests that different antibiotics may trigger distinct pathways leading to enhanced biofilm formation.

Our findings have significant implications for the clinical use of antibiotics and the treatment of biofilm-associated infections. The observation that sub-inhibitory concentrations of fosfomycin enhance *S. aureus* biofilm formation underscores the importance of maintaining appropriate antibiotic levels in clinical practice. This is particularly relevant in situations with poor tissue penetration or during long-term therapies where drug concentrations may fluctuate. The enhanced biofilm formation could potentially lead to persistent or recurrent infections, highlighting the need for careful dosing regimens when using fosfomycin, especially with *S. aureus* biofilms.

However, this study has some limitations. Primarily, the experiments were conducted using *in vitro* models, which may not fully capture the complexity of *in vivo* conditions. Factors such as the host immune response, fluctuations in nutrient availability, and the presence of other microorganisms in polymicrobial infections could clinically influence the impact of fosfomycin on *S. aureus* biofilm formation. To translate these findings into clinical practice, further *in vivo* studies and clinical investigations are warranted. In addition, although our findings indicate that fosfomycin-induced biofilm enhancement is dependent on *SarA*, it remains unclear whether fosfomycin regulates *SarA* directly or through indirect mechanisms. Future studies should employ molecular interaction assays, such as microscale thermophoresis and isothermal titration calorimetry, to investigate this interaction.

In summary, this study unravels the mechanism by which sub-inhibitory concentrations of fosfomycin stimulate *S. aureus* biofilm formation. Fosfomycin treatment enhances PIA and eDNA synthesis in *S. aureus*, potentially contributing to the promotion of biofilm formation.
